# Progress and Challenges Toward the Rational Design of Oxygen Electrocatalysts Based on a Descriptor Approach

**DOI:** 10.1002/advs.201901614

**Published:** 2019-11-27

**Authors:** Jieyu Liu, Hui Liu, Haijun Chen, Xiwen Du, Bin Zhang, Zhanglian Hong, Shuhui Sun, Weichao Wang

**Affiliations:** ^1^ Department of Electronics National Institute for Advanced Materials Renewable Energy Conversion and Storage Center Tianjin Key Laboratory of Photo‐Electronic Thin Film Device and Technology Nankai University Tianjin 300071 China; ^2^ Institute of New Energy Materials School of Materials Science and Engineering Tianjin University Tianjin 300350 China; ^3^ Department of Chemistry School of Science, and Tianjin Key Laboratory of Molecular Optoelectronic Science Tianjin University Tianjin 300072 China; ^4^ State Key Laboratory of Silicon Materials School of Materials Science and Engineering Zhejiang University Hangzhou 310027 China; ^5^ Energy, Materials and Telecommunications Research Centre Institut National de la Recherche Scientifique Varennes QC J3X 1S2 Canada

**Keywords:** oxygen electrocatalysis descriptors, oxygen redox reactions, rational material design

## Abstract

Oxygen redox catalysis, including the oxygen reduction reaction (ORR) and oxygen evolution reaction (OER), is crucial in determining the electrochemical performance of energy conversion and storage devices such as fuel cells, metal–air batteries,and electrolyzers. The rational design of electrochemical catalysts replaces the traditional trial‐and‐error methods and thus promotes the R&D process. Identifying descriptors that link structure and activity as well as selectivity of catalysts is the key for rational design. In the past few decades, two types of descriptors including bulk‐ and surface‐based have been developed to probe the structure–property relationships. Correlating the current descriptors to one another will promote the understanding of the underlying physics and chemistry, triggering further development of more universal descriptors for the future design of electrocatalysts. Herein, the current benchmark activity descriptors for oxygen electrocatalysis as well as their applications are reviewed. Particular attention is paid to circumventing the scaling relationship of oxygen‐containing intermediates. For hybrid materials, multiple descriptors will show stronger predictive power by considering more factors such as interface reconstruction, confinement effect, multisite adsorption, etc. Machine learning and high‐throughput simulations can thus be crucial in assisting the discovery of new multiple descriptors and reaction mechanisms.

## Graphical abstract

The descriptors for the activity of oxygen redox reactions are summarized with special attention being paid to their applications as well as the challenges and outlook of these descriptor‐based approaches.

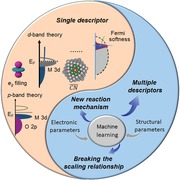

## 1 Introduction

Searching for highly efficient catalysts to promote the sluggish oxygen reactions, namely the oxygen reduction reaction (ORR) and oxygen evolution reaction (OER), plays a pivotal role in various energy storage and conversion devices.^1^ A reasonable descriptor, linking structure and properties, allows experimental specialists to quickly screen materials from the database and rationally optimize the existing catalysts.^2^ In such a way, the cycle of catalysts R&D process could thus be significantly accelerated. However, identifying descriptors and discovering how they determine the activity and selectivity of the electrocatalysts are challenging and remain largely underexplored.

Since the early 20th century, the classical Sabatier principle demonstrates that the best catalysts should bind atoms and molecules with an optimum bond strength: not too weak in order to activate the reactants, and not too strong so as to desorb the products.^3^ However, this principle lacks predictive power because it is not quantitative and cannot be validated experimentally. During the past decades, electronic and structural features, i.e., descriptors, are extracted to characterize the “bond strength” between the relevant intermediates and the catalysts. Typically, the descriptors could be classified into two categories, i.e., bulk‐ and surface‐property‐based descriptors.

For the bulk property based descriptors, *e_g_* filling,^4^ *N*–*V*,^5^ *p*‐band center of bulk oxygen (ε_O−2*p*_),^6^ bulk thermochemistry,^7^ charge‐transfer energy,^8^ etc., have been developed. For oxides, such as spinel, crystal field confinements induce the strong orbital selectivity at the Fermi level. Occupancy of the *e_g_* orbital of octahedral sites could be suitable to describe the catalytic behaviors.[qv: 4a] To predict whether the OER follows the lattice oxygen mechanism (LOM) or the conventional adsorption evolution mechanism (AEM), the *N*–*V* descriptor is essential for describing the activity, stability of oxides.^5^ ε_O−2*p*_, i.e., the position of the bulk O *p*‐band center relative to the Fermi level (*E*_F_), correlates strongly with the oxygen surface exchange kinetics.[qv: 6b] In a similar fashion, the computed ε_O−2*p*_ also correlates with experimental OER activities of cobalt‐based perovskites in alkaline solution.[qv: 6a] Bulk thermochemistry could serve as another descriptor for the OER performance of transition metal oxides (TMOs).^7^ This is because bulk thermochemistry and surface adsorption energetics depend similarly on the number of outer electrons of the transition metal (TM) in the oxide. Normally, a single mechanism is not at play across oxide chemistries. The charge‐transfer energy operates as a descriptor for the OER activity beyond the conventional Sabatier principle involving a single reaction mechanism, and it can rationalize the different mechanistic pathways—electron‐limited transfer, concerted proton–electron transfer (CPET), and proton‐limited transfer—by triggering changes in the critical interfacial properties.^8^

In contrast to bulk property‐based descriptors, the surface ones have received great attention since they could be more intuitive to link to the catalytic properties. Main concentrations have been focused on surface electronic structures, surface atomic coordination, *etc*. *d*‐band theory is widely adopted to predict the volcano relationship of ORR performance versus *ε_d_* (*d*‐band center relative to the Fermi level) of transition metals or alloys.^9^ Based on these findings, numerous efforts have been conducted to continue optimizing *ε_d_* via alloying, doping or interfacing to approach the peak of the volcano.^2^, ^10^ Fermi softness (*S*_F_),^11^ i.e., a weighted summation of the density of states (DOS) of a solid surface, is proposed to quantify and spatially describe the chemical reactivity of solid surfaces.^11^ Different from the *p*‐band center of bulk oxygen, the average O‐*2p*‐state energy (${\bar{\varepsilon }}_{2p}$) is further developed to describe the relationship between the atom‐projected DOS of surface adsorbed oxygen and its ability to form and break bonds with the surrounding metal atoms and hydrogen.^12^

In terms of the atom arrangements on surfaces, the local environment of the active sites matter. For defective catalysts, surface distortion (SD) descriptor is proposed to illustrate the defect contributions to the ORR performance of the PtNi catalysts.^13^ When including the coordination chemistry, the coordinatively unsaturated metal cation ([M_CUS_]) is found to directly correlate with the catalytic performance.^14^ A usual coordination number (cn) is developed to reveal the catalytic behavior of metal catalysts.^15^ Then Calle‐Vallejo et al. initiate generalized coordination numbers ($\bar{\mathrm{CN}}$) as a simple ORR descriptor to identify the active sites on a platinum single crystal, revealing the local structure and chemical environment of active centers.^16^ The bond‐energy‐integrated orbital‐wise coordination number (${\bar{\mathrm{CN}}}^{sd}$), which takes into account both geometrical and electronic structures around the active site, can serve as a simple and accurate descriptor for catalysts consisting of TMOs.^17^

In addition to the abovementioned surface descriptors, from a catalytic reaction point of view, Nørskov et al. proposed computational hydrogen electrode (CHE) model, in which the chemical potential of the (H^+^ + e^−^) pair is related to half of the chemical potential of hydrogen.^18^ Linear relations are identified within the adsorption energies of intermediate species on transition metal as well as non‐metallic surfaces.^19^ Scaling relationships can be used to increase the efficiency of predictions and generate Δ*G*_OH_ and Δ*G_O_* − Δ*G*_OH_ as the descriptor for ORR and OER, respectively.[qv: 19a,20] But sometimes they impose a significant limitation on electrocatalysts design. How to design the active sites that deviate from them is a significant way to further decrease the overpotential.^21^

Although the descriptor‐based approach has made great progress, each descriptor still has its limitations. In this Review, we summarize the benchmark descriptors for designing electrocatalysts, through which we provide an opening for the discussion of the correlations among different descriptors. Understanding the interplay of the electronic structures of the catalysts and the local coordination environment of the active sites helps to correlate the above descriptors together and to obtain more practical and universal ones. Particular emphasis is put on recent advances in breaking the scaling relationship of the reaction intermediates to achieve superior performance. The guiding principle of interface engineering is discussed. In this aspect, multiple descriptors would show stronger predictive power for hybrid materials due to the possible effects that occur at the interface. Finally, we discuss the current issues, applications, and outlook of the development of future descriptors.

## 2 Progress on Descriptor‐Based Approach for Oxygen Redox Reactions

### 2.1 Bulk‐Property‐Based Descriptors

Principles are developed for designing catalysts to promote the oxygen kinetics by identifying connections between bulk electronic structure and surface physiochemical properties. In the past few decades, descriptors including *e_g_* filling,[qv: 4a–c] *N*–*V*,^5^ *p*‐band center of bulk O,^6^ charge‐transfer‐energy,^8^ as well as the bulk thermochemistry,^7^ etc. are established.

#### 2.1.1 *e_g_* Filling

Ternary oxides, such as spinel (AB_2_O_4_),^22^ mullite (AB_2_O_5_),^23^ and perovskite (ABO_3_)[qv: 1b] have attracted tremendous attention due to their superior performance and high stability under alkaline environment. Furthermore, this class of materials provides numerous degrees of freedom for conducting systematic explorations to relate intrinsic physicochemical properties with catalytic behaviors. For these materials, *e_g_* filling is adopted to describe trends of the ORR/OER activity.[qv: 4a–c,24]

*e_g_* filling was first proposed as a bulk‐property‐based descriptor. In the 1970s, Matsumoto et al. proposed the hypothesis that the ORR activity of oxide electrodes can be greatly influenced by the formation and filling of a σ* band between the *e_g_* orbital of bulk transition‐metal ions and a molecular orbital of surface oxygen.[qv: 4d–f] Analogously, recent research demonstrates that the catalytic performance of these oxide catalysts depends on the coordination environment of TM.[qv: 4a,24,25] Crystal field in these catalysts induce strong orbital selectivity at the Fermi level, and the maximum spatial overlap between TM splitting *d*‐orbitals and incoming molecular orbitals would benefit the subsequent catalytic reactions.[qv: 1b] This theory is later applied to spinel and mullite materials.[qv: 4a–c]

**Figure 1:**
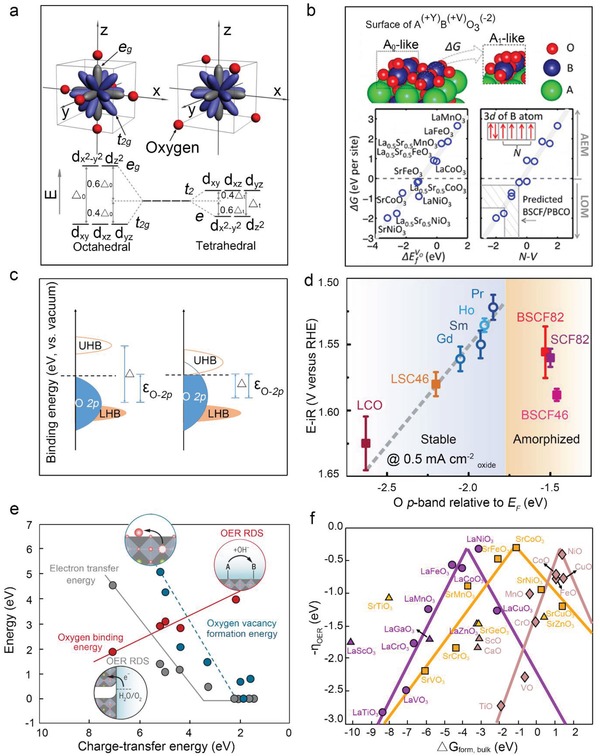
a) Schematic for the spatial arrangement of oxygen ligands in a metal–oxygen octahedral and tetrahedral coordination with respect to five *d*‐orbitals, and the energy levels of the degenerate *d*‐orbitals in octahedral and tetrahedral coordination. Reproduced with permission.[qv: 4a] Copyright 2017, Wiley‐VCH. b) Free energy difference between A_1_‐ and A_0_‐like intermediates versus bulk V_O_ formation enthalpy, relative to H_2_O and H_2_ molecules, and *N*–*V*. Reproduced with permission.^5^ Copyright 2016, American Chemical Society. c) *p*‐Band of bulk O (ε_O−2*p*_) and charge‐transfer‐energy (∆). Relationship between the M *3d* (orange) and O *2p* (blue) band positions. The M *3d* band is shown schematically divided into the upper Hubbard bands (UHB) and the lower Hubbard bands (LHB). Reproduced with permission.^8^ Copyright 2017, Royal Society of Chemistry. d) Evolution of the *iR*‐corrected potential at 0.5 mA cm^−2^_oxide_ versus the O *p*‐band center of (Ln_0.5_Ba_0.5_)CoO_3−δ_ with Ln = Pr, Sm, Gd, and Ho, for LaCoO_3_ (LCO), La_0.4_Sr_0.6_CoO_3−δ_ (LSC46), Ba_0.5_Sr_0.5_Co_0.8_Fe_0.2_O_3−δ_ (BSCF82), Ba_0.5_Sr_0.5_Co_0.4_Fe_0.6_O_3−δ_ (BSCF46), and SrCo_0.8_Fe_0.2_O_3−δ_ (SCF82). Reproduced with permission.[qv: 6a] Copyright 2013, Springer Nature. e) Relationship of charge‐transfer energy to relevant energetics and rate‐determining steps: oxygen vacancy formation (blue), oxygen binding energy (red), and electron transfer energy (gray). Reproduced with permission.^75^ Copyright 2017, AAAS. f) Volcano‐type activity plots for LaMO_3_ (violet circles), SrMO_3_ (orange squares), and MO (brown rhomboids) as a function of the formation energy of the oxide in the bulk. Triangles represent the data for which the linear relationships do not apply. Reproduced with permission.^7^ Copyright 2015, American Chemical Society.

The spinel family of materials contains both tetrahedral and octahedral sites, exhibiting a large degree of cation disorder over the two types of lattice sites.^22^ As displayed in **Figure** **1**a, in octahedral sites, the high‐lying *e_g_* orbital is readily to form a strong spatial overlap with an O‐2*p* orbital. While in tetrahedral coordination, neither the *e* nor *t_2_* orbital directly points to O. The mismatch of the angle between the orbital and the ligand causes weaker spatial overlap with O. By investigating a series of Mn_*x*_Co_3–*x*_O_4_, Xu et al. demonstrated that among two types of crystal fields only *e_g_* filling of octahedral‐coordinated Mn could quantitatively describe the ORR/OER activities.[qv: 4a] For the ORR, if *e_g_* < 1, it is difficult to regenerate OH^−^ due to the too strong M—O^2−^ bond. While *e_g_* > 1, the limiting step is OO^2−^/OH^−^ displacement. As for the OER, too few *e_g_* filling hinders the formation of M—OO,^2−^ and too much *e_g_* filling limits the rate of M—OOH^−^ regeneration. A moderate *e_g_* filling (*e_g_* **≈** 1) at the octahedral site results in the optimization of ORR/OER activity. Analogous to the perovskite family, stronger covalency gives better activity with the same *e_g_* filling. This hypothesis is then applied to explain the ORR/OER activities of other transition‐metal spinels, including Mn_*x*_Co_3−*x*_O_4_ (*x* = 2, 2.5, and 3), Li_*x*_Mn_2_O_4_ (*x* = 0.7 and 1), XCo_2_O_4_ (X = Co, Ni, and Zn), and XFe_2_O_4_ (X = Mn, Co, and Ni). They further investigated the composition dependence of ORR in ZnCo_*x*_Mn_2−*x*_O_4_ (x = 0.0–2.0) with particular attention to the role of edge‐sharing [Co*_x_*Mn_1−_ *_x_*O_6_] octahedra.^25^ Specifically, the ORR specific activity essentially relates to the superexchange interaction (Mn–O–Co) between mixed [MnO_6_]–[CoO_6_] octahedra via the sharing oxygen. As a consequence of the superexchange effects, the *e_g_* filling of active Mn (0.3–0.9) is fine‐tuned when changes the ratio of Co/Mn.

This *e_g_* filling could also apply to Mn‐mullite, which contains two different crystalline fields, i.e., the Mn‐centered pyramid and octahedron.[qv: 4b,26] Density functional theory (DFT) calculations have preliminarily revealed the reasonable performance of mullite SmMn_2_O_5_ in the ORR process, further validated by the experimental measurements in the Mg‐air and Zn‐air battery.[qv: 4c,23,27] The pyramid field (Mn_pyr_^3+^) introduces unit occupancy of the *d_z_^2^* orbital around the Fermi level.[qv: 4b] To further realize the *e_g_* unit filling, Mn^4+^ needs to convert into Mn^3+^. Dong et al. improved ratio of Mn^3+^/Mn^4+^ via introducing oxygen vacancy by laser fragmentation, accompanied with a continuous increase of ORR performance.^28^

In contrast to the bulk‐based hypothesis proposed previously by Matsumoto et al.,[qv: 4d–f] Shao‐Horn and her co‐workers demonstrated the quantitative correlation between the *e_g_*‐filling of perovskite surface TMs and intrinsic ORR/OER activity.^24^ The activity of perovskites has been linked to a number of parameters, including the transition‐metal redox couple,^29^ electrical conductivity,^29^ transition‐metal *d*‐electron count,^30^ transition‐metal *e_g_* occupancy,^24^ metal−oxygen covalency,^8^ σ*‐band filling,^31^ oxidation state,^32^ and O *p*‐band center,^6^ etc. Among the above findings, *e_g_* orbital filling and the metal–oxygen covalency play an important role in governing the activity.^6^, ^24^ *e_g_* orbitals, i.e., *d_z2_* and *d_x2‐y2_*, maximize the *p*–*d* hybridization with intermediates. Achieving *e_g_* unit filling would be the key to obtain optimal ORR/OER performance because of the maximum spatial overlap between the TM site and O*‐containing species. The *e_g_* filling slightly smaller (bigger) than 1.0 results in the optimal ORR (OER) performance.^24^ *e_g_* symmetry of perovskites produces definitive volcano plot with respect to ORR/OER activity.^24^ With *e_g_* filling close to unity as well as higher covalency of metal–oxygen bonds, Ba_0.5_Sr_0.5_Co_0.8_Fe_0.2_O_3−δ_ (BSCF) is found to show superior OER activity in regards to iridium oxide catalyst in alkaline media at least an order of magnitude higher, in spite of the readily amorphization during the OER.[qv: 24b,33] However, the value of *e_g_* filling in their work was estimated from ex situ bulk‐sensitive measurements (i.e., hard X‐ray absorption and magnetometry) or obtained from computed binding energies of oxygenated species on the surface.^32^ In order to validate this electronic structure descriptor, advanced surface‐sensitive techniques remain to be developed for accurately measuring the *e_g_* occupancy of oxides surface during the ORR/OER.

#### 2.1.2 *N*–*V*

Traditionally, theoretical studies assume the OER mechanism evolves via OH*, O*, OOH*, and OO* intermediates bound to surface TMs, that is, the AEM.^34^ In comparison, Kolpak et al. proposed the LOM, in which a surface lattice oxygen shifts out of the surface plane to react with OH* on the TM site to form a surface oxygen vacancy (V_O_) and OO*.^5^ In order to clarify the properties that govern the preferred OER mechanism, in 2016, Kolpak and her co‐workers used ab initio computational schemes to investigate BO_2_‐terminated surfaces of ABO_3_ (A = La, La_0.5_Sr_0.5_, Sr; B = Mn, Fe, Co, and Ni). As shown in Figure 1b, surfaces are initially modeled with one H* adsorbate per unit cell, i.e., 1/4 monolayer. A_0_ has O* on top of 1/4 surface B ions. A_1_ is an isomer of A_0_, of which an unprotonated oxygen is removed from the BO_2_ surface plane and placed on top of O* to form OO* and V_O_. They calculated the free energy difference (Δ*G*) of A_0_‐like intermediate of the AEM and A_1_‐like intermediate of the LOM, relative to H_2_O and H_2_ molecules. The result demonstrates that Δ*G* linear correlates with V_O_ formation enthalpy, $\Delta E_{f}^{V_{0}}$. Moreover, it is found that AEM mechanism dominates when Δ*G* > 0, or vice versa. To be simpler, these two mechanisms could be illustrated by *N*–*V* descriptor, where *N* is the number of unpaired electrons on the isolated B atom and *V* is the nominal valence charge of B in the stoichiometric bulk ABO_3_. *N* is positively correlated to the electropositivity and thus the ability of the B atom (B = Mn, Fe, Co, and Ni) to denote electrons; *V* is the nominal number of donated electrons, and is correlated to the ionization energy for a specific B atom. Thus, *N*−*V* is indicative of net ability to donate electrons, as well as the bond strength between the B‐site and oxygens, corresponding to Δ*G*. LOM is one of the promising schemes to complete the picture of the AEM‐based volcano curve.^35^ Experimentally, Xu et al. demonstrated that OER proceeds via LOM over Zn*_x_*Co_1‐_ *_x_*OOH if two neighboring oxidized oxygens can hybridize their oxygen holes without sacrificing metal–oxygen hybridization significantly.^36^

#### 2.1.3 p‐Band Center of Bulk O

Inspired by the key role of O migrations in solid oxide fuel cells (SOFCs), in 2011, the bulk O *p*‐band center relative to *E*_F_ (ε_O−2*p*_) was proposed as an important descriptor for the activities of perovskites.^6^ This descriptor can be understood through a simple rigid band model as shown in Figure 1c. O addition corresponds to moving electrons from *E*_F_ to the *p*‐band, and vice versa. The Madelung potential and oxygen electron affinity (both are similar in perovskite family) largely determine the absolute energy of the *p*‐band of oxygen. ε_O−2*p*_ thus reflects differences in the Fermi energy of the oxide, which can be adjusted by changing the electronegativity or oxidation state of the B‐site element.^37^

Lee et al. revealed the correlation of ORR catalytic activity with the calculated ε_O−2*p*_ in electron‐rich perovskites in SOFCs.[qv: 6b] Analogous to *d*‐band theory, perovskites with neither too low nor too high O *p*‐band center possess both considerable activity and stability. Grimaud et al. demonstrated that pseudocubic and double perovskites with ε_O−2*p*_ very close to *E*_F_ exhibit the highest activities for both OER and surface oxygen exchange kinetics upon oxygen reduction at elevated temperatures (Figure 1d).[qv: 6a]

Decreasing the O *2p* band center and increasing the overlap between the occupied O *2p* valence band and the unoccupied TM *3d* conduction bands would enhance the OER activity.^38^ However, DFT calculations at the local density approximation (LDA) or generalized gradient approximation (GGA) level are known to produce errors describing the energetics of TMOs.^39^ It is difficult to treat electron correlations in late transition‐metal states of oxides based on DFT without experimental input.^40^ DFT + U method is often used to account for on‐site correlation in the *d* electrons of the transition metal atoms.[qv: 39a] However, the choice of U in the DFT calculations that artificially splits the occupied and unoccupied TM *3d*‐states apart and affects the calculated O *2p*‐band center. Experimental measurements of the O‐2*p* PDOS are thus essential to validate this electronic‐based descriptor. By combining information from X‐ray emission, absorption, and photoelectron spectroscopy, the occupied and unoccupied band positions relative to the oxide Fermi level on an absolute energy scale could be determined. The experimental trend in the O 2*p*‐band center is in good agreement with that obtained from both unoccupied and occupied PDOS from DFT calculations, but **≈**1 eV higher.^8^

#### 2.1.4 Charge‐Transfer Energy

The conventional OER mechanism, i.e., the four CPET steps, might not be the single governing mechanism across oxide chemistries.^8^ A more complex interplay exists between the oxide electronic structures and their catalytic activities. The charge‐transfer energy (Figure 1c) is defined as the relative energies of TM 3*d* and O 2*p* valence electronic states, which acts as a key property for describing the OER activity to relate the bulk electronic structure and surface properties of perovskite oxides. Different from the adsorbate binding perspective, this work provides new insights into the OER descriptors.

Synchrotron soft X‐ray emission and absorption spectroscopy are adopted to validate the above assumptions via examining the metal and oxygen partial DOS near the Fermi level for perovskite oxides. As shown in Figure 1e, when charge‐transfer energy is decreased, the rate‐limiting step transitions from electron transfer‐limited, to proton–electron‐coupled, then to proton transfer‐limited, a trend that holds across a wide breadth of chemistries ranging from insulators to metals. A Marcus model is applied to verify the potential energy surfaces for sequential and CPET pathways.

#### 2.1.5 Bulk Thermochemistry

Experimental electrocatalysis has traditionally resorted to bulk thermochemistry to describe activity trends.^41^ Calle‐Vallejo et al. explained why it can be a good descriptor for the OER electrocatalysis on the basis of transition‐metal perovskites and monoxides.^7^ The linear correlation is identified between the surface adsorption and bulk thermochemistry of SrMO_3_, LaMO_3_, and MO, where M is a metal from Sc to Ge, except those TMOs such as CaO, LaScO_3_, and SrTiO_3_ that possess noble gas configurations. This bulk–surface scaling relationship generates because both surface and bulk properties are dependent on the number of outer electrons. The concept of outer electrons explains the existence of scaling relationships between adsorbates on TMs, their monoxides and ternary oxides in the perovskite phase,^32^, ^42^ and in functionalized graphitic materials and metalloporphyrins with transition‐metal centers.^43^ The correlation yields volcano curves that rationalize the trends in catalytic activity as a function of bulk thermochemistry. As shown in Figure 1f, the volcano curves demonstrate that the majority of the oxides lie on their left legs, thus the most active compounds tend to be the least stable ones. Outer electrons thus provide simple rationales to predict new oxide catalysts.

### 2.2 Surface‐Property‐Based Descriptors

Catalytic reactions occur on the catalyst surface, on which the active sites are sensitive to the local environmental conditions. In contrast to bulk physical and chemical properties, the surface involves into the reactions directly. It tends to be more complicated to link the surface features and the adsorption behavior of the reaction intermediates. The surface electronic structure information and the coordination chemistry should be fully taken into consideration. To date, from electronic structure point of view, *d*‐band theory,^9^ Fermi softness,^11^ the average O‐2*p*‐state energy (${\bar{\varepsilon }}_{2p}$) are proposed.^12^ Considering the coordination chemistry, SD,^13^ coordinatively unsaturated metal cation ([M_CUS_]),^14^ generalized coordination numbers ($\bar{\mathrm{CN}}$),^16^ and the bond‐energy‐integrated orbital‐wise coordination number (${\bar{\mathrm{CN}}}^{sd}$) are more appropriate to act as descriptors.^17^ In the following, we review the achievements during the last several decades of the surface descriptors including the theoretical foundation, applications, and their possible limitations.

#### 2.2.1 *d*‐Band Theory

**Figure 2:**
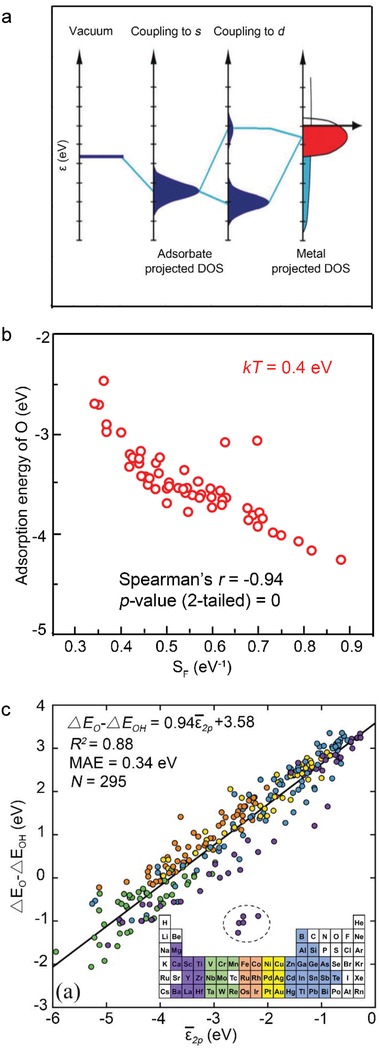
a) Schematic illustration of the formation of a chemical bond between an adsorbate valence level and the *s‐* and *d*‐states of a transition‐metal surface. Reproduced with permission.^76^ Copyright 2011, National Academy of Sciences. b) The correlation between adsorption of O and *S*_F_ when *kT* = 0.4 eV. Reproduced with permission.^11^ Copyright 2016, Wiley‐VCH. c) Correlation between ${\bar{\varepsilon }}_{2p}$ and reactivity of surface oxygen atoms adsorbed at various metal and metal‐oxide surfaces. The coloring of the points is related to the group of the metal that O is bound to as indicated in the inset periodic table. Reproduced with permission.^12^ Copyright 2019, Elsevier.

Back to the 1960s, Newns–Anderson model was proposed to describe the electronic structure determination of the adsorbate to metal surfaces,^44^ upon which Hammer and Nørskov developed *d*‐band theory. The basic idea results from the dependence of the binding energy of an adsorbate to a metal surface on the electronic structure of the surface itself.^9^ When oxygen intermediates, such as O*, OH*, OOH*, OO* react on the catalyst surface, the localized *d*‐band center governs the bonding strengths of the adsorption species with the catalyst surface. As shown in **Figure** **2**a, the formation of bonds between *d* orbital and oxygen *p*‐orbitals gives rise to two states, i.e., the filled bonding states and the partially occupied antibonding states. The increased fraction of unoccupied antibonding states causes the strengthening of adsorbate‐surface interaction with a higher *d*‐band center and vice versa.^2^ Refinements, e.g., *d*‐bandwidth, have been developed to make the *d*‐band theory more accurate to describe O* adsorption from one metal to the next.^45^

*d*‐Band theory has been extensively applied in TMs and alloys. Pt is so far the most efficient single metallic electrocatalyst due to its appropriate *ε_d_* position. However, it still remains room for Pt to continue improving its ORR performance.^18^ 0.0–0.4 eV more weakly metal–oxygen bond strength than Pt should possess higher activity.^18^, ^46^ Various strategies, such as alloying, straining, and the substrate supporting have been carried out to weaken the metal–oxygen bondings.[qv: 2,10a,b,47] Pt_3_M (M = Ti, V, Cr, Mn, Fe, Co, and Ni) intermetallic compounds could lower *ε_d_* of the surface Pt‐skin layer, and thus decrease the oxygen binding energy.[qv: 10b,48] Considering the smaller electronegativity of Sc and Y, they donate electrons to the *d*‐orbitals of Pt and thus reduce *ε_d_* of Pt. Experimentally, polycrystalline Pt_3_Sc and Pt_3_Y are confirmed as more efficient and stable electrocatalysts than pure Pt for ORR, with the activity enhancement of 1.5–1.8 and 6–10 times, respectively.^46^ The Pt_3_Ni(111) surface is tenfold more active than the corresponding Pt(111) surface and 90‐fold more active than the Pt/C catalysts. The *d*‐band center of the surface Pt‐skin is downshifted of about 0.34 eV compared to pure Pt single crystals.^49^ Substrate Ti_0.7_Mo_0.3_O_2_, tin‐doped indium oxide, and 2D Ti_3_C_2_X_2_ (X = OH and F) nanosheets, are recently investigated to replace the commonly used carbon black to optimize *ε_d_* for better stability and activity.^50^ *d*‐band theory acts as one of the excellent descriptors to handle many interesting reactions. Nevertheless, it fails to embrace catalyst surface information and invalids for non‐metallic systems.[qv: 6b]

#### 2.2.2 Fermi Softness

To overcome the weakness of *d*‐band theory which fails to provide spatial distribution information, Huang et al. developed a descriptor named “Fermi softness” (*S*_F_) for describing the electronic structures of a solid surface.^11^ The contributions of different states in the band are unequal to surface bonding. The closer ones to the Fermi level make a greater contribution, determined by a reactivity weight function, i.e., the derivative of the Fermi‐Dirac distribution function (a weight function, donates as *w(E)*). The spreading of the *w(E)* can be adjusted with the parametric temperature (*T*) or the nominal electron temperature (*kT*, where *k* is Boltzmanns constant). When *kT* is set to a non‐zero value, 0.4 eV for example, the resulting *S*_F_ becomes significantly correlated with the surface reactivity as illustrated in Figure 2b.

*S*_F_(*r*) has been validated by various systems such as metals, alloys, and even complicated compounds (1D MoS_2_ edge and N‐doped graphene). Take O* adsorption on Pt_3_Y(111) surface as an example, *S*_F_ successfully explains the binding strength of Pt—O is stronger than that of Y—O, even if the *d*‐band center of Y is higher than that of Pt on the surface. Based on *S*_F_, Zhang et al. further proposed Fermi‐abundance descriptor through the investigation of the adsorption of H atom on defective monolayer VS_2_ and MoS_2_.^51^

#### 2.2.3 Average O‐2p‐State Energy (${\bar{\varepsilon }}_{2p}$)

By examining the reactivity and electronic structure of adsorbed oxygen, rather than the metal atom to which it binds, Dickens and his colleagues proposed the average O‐*2p*‐state energy (${\bar{\varepsilon }}_{2p}$) as the descriptor to illustrate the metal–O coupling strength.^12^ ${\bar{\varepsilon }}_{2p}$ is calculated as the first moment of the DOS projected onto the atomic *2p*‐states of an adsorbed oxygen atom relative to the Fermi level. It is defined as Equation (1):

(1) $${\bar{\varepsilon }}_{2p}=\frac{\;\int_{\varepsilon_{\min}}^{\varepsilon_{\max}}\;\rho_{2p}\varepsilon d\varepsilon }{\int_{\varepsilon_{\min}}^{\varepsilon_{\max}}\;\rho_{2p}d\varepsilon }$$

The oxygen *2p*‐states usually lie above −10 eV relative to the Fermi level, as a result, this value is chosen as the lower energy bound. While ε_max_ value is in the range of 1–2 eV relative to the Fermi level.

As shown in Figure 2c, the data points consist of face‐centered‐cubic metal, (111) surfaces, rutile metal‐oxide (110) surfaces, and cubic perovskite (100) surfaces. Generally, Δ*G*_O_ − Δ*G*_OH_ is the conventional activity descriptor for OER,^2^ which is in consistent with Δ*E*_O_ − Δ*E*_OH_. In this work, for each point, ${\bar{\varepsilon }}_{2p}$ of a specific oxygen atom is compared to the independently calculated Δ*E*_O_ − Δ*E*_OH_ for the same oxygen atom. The data set as a whole has a near‐unity slope, indicating the close correlation with Δ*E*_O_ − Δ*E*_OH._

#### 2.2.4 Surface Distortion

By exploring the catalytic performance of various different PtNi nanocatalysts, SD is put forward as a structural descriptor to capture the degree of surface defectiveness.^13^ Chattot et al. experimentally identified that the density of bulk defects (e.g., chemical disorder) is governed by the Ni content and the compensation of surface defectiveness (such as grain boundaries and surface dealloying) by the bulk is determined by the surface‐to‐volume ratio. Based on these facts, Equation (2) is established:

(2) $$\mathrm{SD}\;=\frac{\mathrm{Microstrain}-f\left(\mathrm{Ni}\%\right)}{D}$$

where “Microstrain” is the microstrain value derived from Rietveld refinement of the synchrotron wide‐angle X‐ray scattering patterns, *f*(Ni%) is the bulk contribution induced by inhomogeneous alloying directly linked to the average Ni content in the catalyst, and *D* is the dispersion or “surface atoms ratio”.

**Figure 3:**
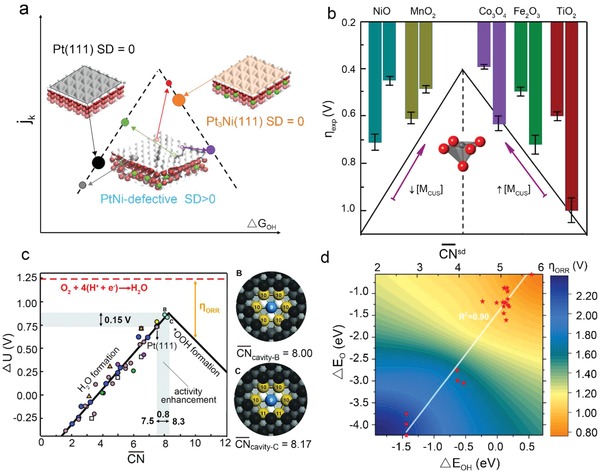
a) The volcano curve between SD and the kinetic current (*j_k_*). The scattergun approach (SD > 0) versus the homogeneously optimized surface approach (SD = 0) for structurally disordered and structurally ordered catalysts, respectively. Reproduced with permission.^13^ Copyright 2018, Springer Nature. b) The overpotential dependence on surface M_[CUS]_ for TMOs. Reproduced with permission.^14^ Copyright 2016, American Chemical Society. c) Coordination‐activity plot for metallic catalysts. Potentials for the two limiting steps on extended surfaces and nano‐particles. B and C points correspond to the right two structure where $\bar{\mathrm{CN}}$ ≈ 8.0 and 8.17, respectively. Reproduced with permission.^16^ Copyright 2015, AAAS. d) ORR theoretical limiting potential plot. The ORR overpotential as a function of ${\bar{\mathrm{CN}}}^{sd}$. The relationship of adsorption energy of O* and OH* as well as ${\bar{\mathrm{CN}}}^{sd}$. Reproduced with permission.^17^ Copyright 2018, American Chemical Society.

As displayed in **Figure** **3**a, the Sabatier plot between the kinetic current (*j_k_*) and the DFT calculated hydroxyl binding energy (Δ*G*_OH_) is established. In the “structurally ordered” family (SD = 0), take the Pt_3_Ni(111) surface for example, all the catalytic sites uniformly target the orange circle. In contrast, “structurally disordered” catalysts (SD > 0) feature a wide distribution of catalytic site configurations. And those nearest to the top of the volcano most likely determine the global reaction rate.

#### 2.2.5 Coordinatively Unsaturated Metal Cation

Coordinatively unsaturated metal cation (M_CUS_) is developed from molecular orbital and solid state band structure principles, which describes chemisorption strength by the relative occupancy of antibonding states.^14^ Compared to the *d*‐band theory which clarifies that the adsorption energy variations on TMs are mainly contributed from the interaction of adsorbates with *d*‐states of metal atoms, the energy of highest occupied *d*‐states relative to the Fermi level (denoted as *E_d_*) determine the bond strength of adsorbates on TMOs.

Tao et al. systematically investigated the OER catalytic performance of some binary oxides as shown in Figure 3b. They clarified that weak‐binding TMOs would benefit from higher [M_CUS_]. For example, the OER activity of TiO_2_ with higher [M_CUS_] is significantly improved in terms of a combined evaluation of onset potential, Tafel slope (51 mV dec^−1^), Faradaic efficiency (100%), and stability. For n‐type semiconductors such as TiO_2_, *E_d_* is far below the Fermi level, the antibonding states are totally occupied, leading to weak bonding. By creating M_CUS_ on the surface, mid‐gap states appear near the Fermi level due to unpaired *d*‐electrons. The antibonding states are partially filled thus strengthening the adsorption. For p‐type TMOs (i.e., MnO_2_, Co_3_O_4_, and NiO), *E_d_* is much closer to *E*_F_. When introducing more [M_CUS_], further upshift of *E_d_* gives rise of stronger bonding as compared to the stoichiometric p‐type TMOs. The OER overpotential difference for each TMO with lowest to highest [M_CUS_] is summarized in Figure 3b, which indicates that M_CUS_ leads to a vast difference in OER kinetics. Therefore, the adsorption energy can be tailored rationally for TMOs in OER.

#### 2.2.6 Generalized Coordination Numbers

To reveal the local structure and chemical environment of active centers, as shown in Figure 3c, Calle‐Vallejo et al. presented “generalized” coordination numbers ($\bar{\mathrm{CN}}$) as a descriptor that predicts the geometric structure of optimal active sites.^16^, ^52^ For TMs, $\bar{\mathrm{CN}}$ for site *i* is described as follows:

(3) $$\bar{\mathrm{CN}}\;\left(i\right)=\sum_{j=1}^{n_{i}}\frac{\mathrm{cn}\left(j\right)}{{\mathrm{cn}}_{\max}}$$

where *n_i_* is the coordination number (cn) of site *i*, around this site, one finds the first‐nearest neighbors *j*, cn(*j*) is the coordination number of atom *j*, and cn_max_ is the maximum number of first‐nearest neighbors in the bulk. By using $\bar{\mathrm{CN}}$ as the descriptor, they predicted the optimal ORR active sites on Pt possess $\bar{\mathrm{CN}}\approx 8.3$, which is verified by the high ORR performance of the experimentally constructed active sites at Pt(111) with $\bar{\mathrm{CN}}\approx 8$. They also extended the feasibility of $\bar{\mathrm{CN}}$ on other types of materials (e.g., alloys, *3d*‐TMOs, perovskite oxides, etc.) by introducing more factors such as bond strengths, oxidation state effects, and orbital splitting effects.[qv: 43b]

#### 2.2.7 Bond‐Energy‐Integrated Orbital‐Wise Coordination Number (${\bar{\mathrm{CN}}}^{sd}$)

In terms of the coordination of the active sites on the surface, Wu et al. proposed a bond‐energy‐integrated orbital‐wise coordination number as an effective descriptor for TMOs.^17^ ${\bar{\mathrm{CN}}}^{sd}$ is derived based on the moments theorem of the local density of states (LDOS) proposed by Cyrot–Lackmann:

(4) $${\bar{\mathrm{CN}}}^{sd}=\frac{\sum_{i\neq i^{\prime }}\sqrt{\mu_{2,i}^{sd}}}{\sqrt{{\left(V_{nn}^{sd,\infty }\right)}^{2}}}$$

where $\mu_{2,i}^{sd}$ is the second moment for the LDOS of *s* and *d* orbitals projected onto an active site *i* in the surface. $V_{nn}^{sd,\infty }$ is the sum of the square of the *s* and *d* electron hopping integrals to a neighboring atom in bulk. ${\bar{\mathrm{CN}}}^{sd}$ involves the contribution of the bond energy of *s* and *d* orbitals, which is embodied by the square root of the second moment for the LDOS.

The adsorption energies of OOH* and O* have a strong linear correlation with ${\bar{\mathrm{CN}}}^{sd}$ over various β‐MnO_2_ surfaces such as (111), (210), (310), and (100). In contrast, the linear relationship is weaker between cn and the adsorption energies. Meanwhile, the root‐mean‐square error (RMSE) for cn is larger than that of ${\bar{\mathrm{CN}}}^{sd}$. Figure 3d presents the correlation between overpotential (η) and the adsorption energies of *O and *OH (Δ*E*_O_ and Δ*E*_OH_) for β‐MnO_2_. Obviously, an ideal β‐MnO_2_ catalyst ought to have a high ${\bar{\mathrm{CN}}}^{sd}$ located at the right upper corner of Figure 3d. This design principle is also applied to the archetype OER catalyst, RuO_2_. The fitting data of the adsorption energy of OH* with ${\bar{\mathrm{CN}}}^{sd}$demonstrates that *R* ^2^ and RMSE values are 0.85 and 0.12, respectively, displaying a satisfied scaling relationship.

Except for the surface descriptors mentioned above, the OH—M^2+δ^ bond strength,^53^ the descriptor for doped‐graphene,^54^ etc. are developed. Each descriptor is suitable for the specific system. The underlying physics should drive more attention to discover universal ones for the future design of electrocatalysts.

## 3 Recent Developments on the Descriptor Approach for Oxygen Electrocatalysis

### 3.1 Strategies to Break the Linear Scaling Relationships to Enhance ORR/OER

#### 3.1.1 Origin of the Linear Scaling Relationships

Nørskov first initiated CHE method to describe the free energies of the reactive intermediates for oxygen redox reactions thermodynamically.^18^ In general, the CPET steps accompany with the adsorption and desorption of the intermediates, such as O*, OH*, and OOH*.^55^ The adsorption energies of the intermediates obey a linear scaling relationship between one another,[qv: 15,19b,42]

(5) $$\Delta G_{2}^{\left(i\right)}=\;A_{1,2}^{\left(i\right)}\cdot \;\Delta G_{1}^{\left(i\right)}+\;B_{1,2}^{\left(i\right)}$$

where $\Delta G_{1}^{\left(i\right)}$ and $\Delta G_{2}^{\left(i\right)}$ are the chemisorption energies of oxygen intermediates. $\Delta G_{1}^{\left(i\right)}$ and $\Delta G_{2}^{\left(i\right)}$ scale with each other and depend on several structural parameters {ω_*i*_} as $\Delta G_{1}^{\left(i\right)}=f^{i}\;(\{\omega_{i}\})+\beta_{1}^{i}$, $\Delta G_{2}^{\left(i\right)}=f^{i}\;(\{\omega_{i}\})+\beta_{2}^{i}$, then:

(6) $$f^{i}\;\left(\left\{\omega_{i}\right\}\right)=A_{1,2}\;g^{i}\left(\left\{\omega_{i}\right\}\right)$$

(7) $$B_{1,2}^{\left(i\right)}=\beta_{2}^{i}\;-A_{1,2}\beta_{1}^{i}$$

Fundamentally, the slope of Equation (5) on close‐packed and low‐index surfaces depends strongly on the number of valence electrons of the atoms bound to the surface. The slope between the adsorption energies of *OH and *O is about 1/2, as the oxygen atom in OH* needs one electron to fulfill the octet rule, and O* needs two. The offset depends on the facet and the adsorption site for sets of adsorbates.^56^ Su et al. discovered that several more factors including outer electrons, work function, excess charge, *d*‐band center, integrated crystal orbital overlap population, integrated crystal orbital Hamilton population could influence the structure parameter {ω_*i*_}.^57^

**Figure 4:**
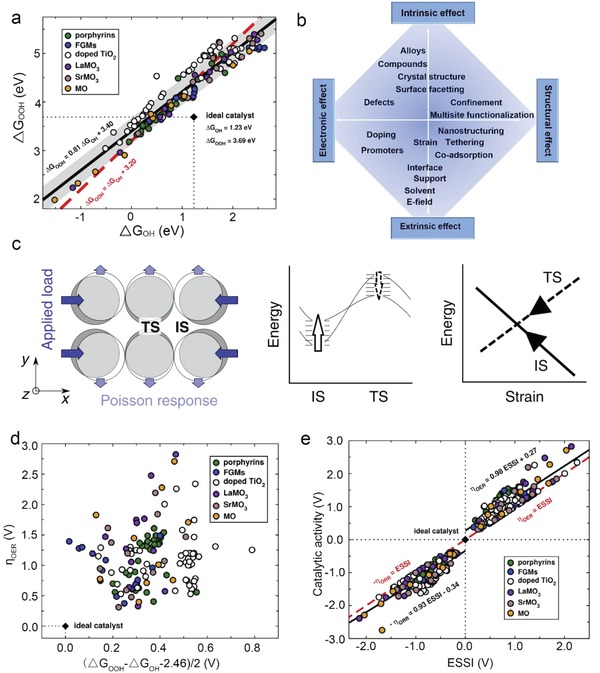
a) Linear relationship between the adsorption energy of OH* and OOH* over various materials. Reproduced with permission.^65^ Copyright 2018, Elsevier. b) Approaches that might be used as possible routes to circumvent energy scaling relations. Reproduced with permission.^21^ Copyright 2015, Oxford University Press. c) Applying uniaxial compression to the surface leads to the opposite response of the initial and transition states. Reproduced with permission.^63^ Copyright 2018, Springer Nature. d) Calculated OER overpotentials for various electrocatalysts plotted as a function of their departures from the ideal ∗OOH versus ∗OH energetic separation of 2.46 eV. Reproduced with permission.^65^ Copyright 2018, Elsevier. e) The catalytic performance as a function of ESSI. Reproduced with permission.^65^ Copyright 2018, Elsevier.

Scaling relationships open the possibility of mapping the many parameters determining the rate of a full catalytic reaction onto a few descriptors. This linear relationship intimately connects with the Sabatier principle and derives the volcano‐shape curves. In many cases, $\Delta G_{\mathrm{OH}}$ and $\Delta G_{O^{\;}}-\Delta G_{\mathrm{OH}}$ are considered as the descriptors for ORR and OER, respectively.^55^, ^58^ Statistical results show that the free energy difference (offsets in Equation (5)) between Δ*G*_OH_ and Δ*G*_OOH_ is 3.2 ± 0.2 eV with a slope **≈**1 for monodentate absorbates (**Figure** **4**a).[qv: 19a] However, the ideal electrocatalyst with zero overpotential would require the energy difference to be twice of the equilibrium potential, i.e., 2.46 eV. Thus, for both ORR and OER, the predicted lowest overpotential is (3.2–2.46) eV/2e ≈ 0.4 V. This prediction is a general phenomenon between any set of adsorbates bound similarly to the surface. Promising strategies are explored to overcome these limitations to usher in a new era of theory‐driven catalyst design. Schemes such as interfacing, doping, strain, etc., would in principle break the linear relations to further reduce the overpotentials (Figure 4b).^21^

#### 3.1.2 Multisite Adsorption to Break the Linear Scaling Relations

So far, pioneers have been dedicated to breaking the scaling relationships both theoretically and experimentally. For instance, single‐atom Au decorated NiFe layered double hydroxide (LDH) shows a sixfold activity enhancement for OER compared to pure LDH.^[59]^ With the assistance of Au single atom, monodentate OOH* transforms into bidentate ones. The DFT calculated overpotential decreases to 0.18 V, due to charge redistribution of active Fe site as well as its surrounding atoms causing by the neighboring single‐atom Au on NiFe oxyhydroxide. The integrated charge density difference yields a net Au‐to‐LDH charge redistribution of 0.32 e, which transfers to surrounding O, Ni, and Fe atoms. The charge redistribution facilitates the adsorption of OH* and modify the adsorption energies of O* and OOH* intermediates, resulting in low overpotential in the rate‐limiting step from O* to OOH*.

**Figure 5:**
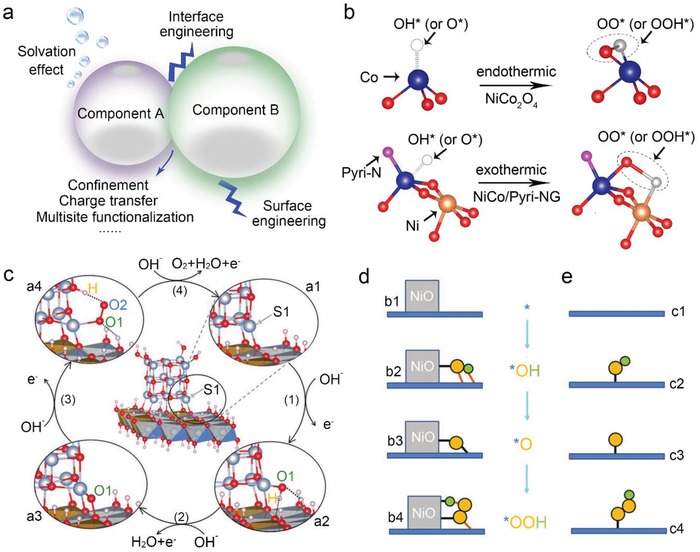
Interface engineering for hybrid materials. a) The possible effects caused by the interface when the hybrids are under operating conditions. b) Adsorption configurations of oxygen‐containing species on the Co site of NiCo_2_O_4_ and NiCo/Pyri‐NG. Color scheme: red for oxygen, blue for Co, orange for Ni, pink for N, and gray for adsorbed O*/OH*. b) Adapted with permission.^68^ Copyright 2018, Wiley‐VCH. c,d) Engineering of 3D active sites at interfaces. e) 2D image of traditional single‐site adsorption on a planar surface. c–e) Reproduced with permission.^60^ Copyright 2019, Wiley‐VCH.

The strategy mentioned above has a starting point that an additional active site is engineered to attract the hydrogen atom in the *OOH intermediate. As a result, multisite functionalization approach could potentially change the adsorption behaviors of the intermediates, thus regulating the rate‐determining steps to enhance the electrocatalysis. Catalysts with several types of active sites or local binding environments for different intermediates could possibly achieve multisite adsorption, i.e., providing a 3D active site including co‐adsorbing or tethering molecules. As shown in **Figure** **5**c, the intermediates are anchored by S1 and surface ligands in NiO and/or NiFe LDH simultaneously, forming tridimensional adsorption which varies dynamically with the type of intermediate in contrast to the traditional single‐site adsorption (Figure 5d,e).^60^

#### 3.1.3 Strain Schemes to Avoid the Linear Scaling Relationships

Applying strain is another efficient way to avoid the scaling relationship. By the *d*‐band model, we can get the general conclusion that compressive strain leads to weaker binding and vice versa.[qv: 9b] However, guidelines are complicated for the variation of surface reactivity with strain, depending on the adsorbate chemical species,^61^ the metal surface species, the reaction site, and the crystal orientation of the surface.^62^

Specifically, the species adsorbed on the surface either “push” the adjacent atoms outward (positive eigenstress) or “pull” them inward (negative eigenstress), inducing a compression or tension in the neighboring region, which can be relieved by applying an external expansive or compressive strain, respectively. The binding energy response to strain depends on the coupling of the adsorbate‐induced eigenstress with the applied strain.^63^ When stress is applied to a catalyst surface uniaxially, the strain itself can be engineered to break the response of adsorbate and transition‐state scaling relations, as shown in Figure 4c. By examining a typical of catalysis: the dimerization of two N adsorbates to form N_2_ over Pt(100) surface, the prediction is verified. The compressive strain from uniaxial loading again causes a deviation from the transition‐state scaling trends.

Analogously, Xie et al. compared the ORR performance on active sites of N‐doped graphene, i.e., the nearest‐neighboring carbon atoms around the doped N, by applying tensile or compressive strain.^64^ The tensile strain tends to stretch and even break the N—C* bond, enhancing the O* adsorption while leaving that of OH* and OOH* approximately unchanged. The projected DOS under different tensile strain show that a σ bond forms in all the three intermediates (O*, OH*, and OOH*). For adsorption of O*, the antibond peak shifts leftward with increasing local strain, indicating the weakened N—C* bond. However, for OH* and OOH*, both the position and the extent of anti‐bond peak nearly remain the same. As a result, the O* adsorption can be controlled individually. The 1D sine‐like rippling graphene model is constructed to study the effect of curvature.

#### 3.1.4 Post‐Treatment after Breaking Linear Scaling Relations

Breaking of adsorption‐energy scaling relationships between OH* and OOH* cannot guarantee the performance enhancement of electrocatalysis, it is a necessary yet insufficient condition to optimize active sites (Figure 4d,e). Govindarajan et al. proposed a new descriptor, the electrochemical‐step symmetry index (ESSI), to ensure the improvement of the catalytic performance after circumventing the linear relations. The descriptor is defined as the following Equation (8), 65

(8) $$\mathrm{ESSI}\;=\frac{1}{n}\;\sum_{i}^{n}\left(\Delta G_{i}^{*}-E^{0}\right)$$

where $\Delta G_{i}^{*}$ is the reaction Gibbs free energy of the elementary reaction, *E* ^0^ is the equilibrium potential, and *n* is the number of Δ*G_i_* that ≥ *E* ^0^. Basically, ESSI emphasizes that catalyst optimization should not only focus on breaking the scaling between OH* and OOH*. For example, in the case of Au functionalized graphitic materials, although the free energy difference between OH* and OOH* is 2.49 eV, the overpotential is 1.49 V.[qv: 43a] Instead, the minimal deviation from the equilibrium potential of the reaction energies of all electrochemical steps guarantees high‐performance electrocatalysis.

In Figure 4e, for different materials include metal and oxides, the ESSI versus the overpotential η_OER_ for the OER and −η_ORR_ for the ORR display a best‐fit lines have slopes close to 1, but the offsets differ by 0.27 + 0.34 = 0.61 V. This agrees well with the double volcano activity plot: the ORR and OER activity summits locate at different adsorption energies.^66^

It is noted that the above discussion is based on the same reaction mechanism. When considering the change of the reaction process and the solvation effect, the story might change. For instance, when the ORR proceeds through the dissociate mechanism, O_2_ splits into 2O* without prohibitive kinetic barriers, the formation of OOH* is avoided and escapes the scaling between OOH* and OH*.

### 3.2 Multiple Descriptors for Hybrid Materials

In comparison with the single‐phase electrocatalyst, mixed‐phase catalysts gain great attention as other phases could effectively tune the electronic structures of the main catalyst and vice versa via the interface engineering. Consequently, the catalytic performance could be promoted through the so‐called synergistic effect. The promotion could be achieved through confinement, multisite adsorption, charge transfer, etc. (Figure 5a).

For instance, N‐doped graphene and spinel NiCo_2_O_4_ along are two oxygen electrocatalysts.[qv: 4a,67] Growth of NiCo_2_O_4_ on pyridinic‐N modified graphene (donate as NiCo/Pyri‐NG in Figure 5b) results in an improvement of the catalytic performance via forming the N—Co bonds.^68^ The DFT calculation result demonstrates that upon pyridinic N doping, Co—N bond (1.98 Å) forms as electrons transfer from Co to N. Consequently, pyridinic‐N, surface Co and its neighboring Ni synergistically promote the efficiency of both ORR and OER processes. As shown in Figure 5b, the as‐formed OO* and OOH* co‐adsorb on Co and its neighboring Ni. The bond of pyridinic‐N and Co leads to an overpotential difference Δ*E* (defined as the difference between the potential required to deliver an OER current density of 10 mA cm^−2^ and the ORR half‐wave potential) among the lowest values for highly efficient bifunctional catalysts.

Similarly, for NiO clusters supported on NiFe LDH,^60^ compared to its single component NiO or NiFe LDH, the NiO/NiFe LDH on Cu foam achieves an overpotential of 205 mV at reaction current 30 mA cm^−2^, with the Tafel slope of 30 mV dec^−1^. At the NiO/NiFe LDH interface (Figure 5c,d), different intermediates are stabilized by different chemical or hydrogen bonds over the intersection, varying dynamically during the whole OER process. The calculated overpotential is 0.2 V, which is out of the volcano curve restricted by the scaling relationship.

Mn‐based mullite oxide AMn_2_O_5_ (A = lanthanide) emerges recently as an efficient ORR catalyst. Zhao et al. synthesized mixed perovskites AMnO_3_ (A = Ca, Sr, Ba) by facile one‐step co‐precipitation method.[qv: 4c] Each mixture includes three phases, i.e., mullite SmMn_2_O_5_, O‐deficient perovskite AMnO_3−δ_, and MnO*_x_*. Atomic bonding interfaces are formed between SmMn_2_O_5_ and AMnO_3−δ_, based on the observations of the high‐resolution transmission electron microscopy. Among these different mixed‐phase samples, Ba*_x_*Sm_1−_ *_x_*Mn_2_O_5−δ_/C exhibits the best ORR catalytic activity with the half‐wave potential **≈**0.79 V (versus RHE) and the highest stability over 20 000 s. Fundamentally, when n‐type perovskite comes to contact with p‐type mullite, majority carriers on each side transfer into the other side through the depletion region. Therefore, this enhanced performance can be ascribed to the large charge transfer from BaMnO_2.83_ to SmMn_2_O_5_ since partial Mn^4+^ in mullite SmMn_2_O_5_ phase are reduced to active sites Mn^3+^ to achieve the *e_g_* unit occupancy in the interfacial depletion region.

More similar works could be found about the mixed‐phase catalysts.^53^, ^69^ Although the catalytic performance is indeed improved through such strategies, it is difficult to propose a descriptor to illustrate the structure–property relations. To address such complicated issues, determination of the interface structure is the first step based on the current advanced experimental characterizations combining with theoretical simulations. Therefore, the occupation and degeneracy of electronic orbitals near the interface could thus be gained. Based on these characterizations, combining the complicated characterized structural and electronic information, discovery of the multiple descriptors for these systems could be urgently needed.

### 3.3 Multiple Descriptors

**Figure 6:**
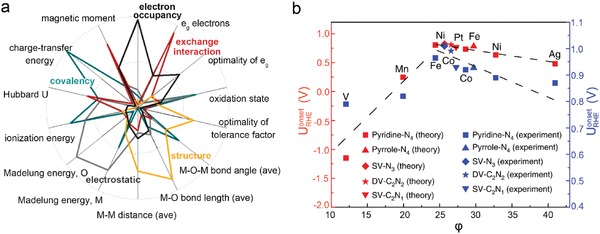
a) Loading magnitudes for the 14 descriptors obtained by factor analysis. Larger radial component indicates a larger contribution of a descriptor to the factor. Reproduced with permission.^70^ Copyright 2016, American Chemical Society. b) Theoretical and corresponding experimental onset potentials for ORR versus the descriptor φ. Reproduced with permission.^71^ Copyright 2018, Springer Nature.

The bulk and surface descriptors mentioned above are normally effective for some specific material systems and reactions. In other words, one descriptor is challenging to deal with different catalysts generally. Importantly, with the assist of the machine learning and high‐throughput calculations, multiple descriptors begin to be proposed for the description of the reactions. As shown in **Figure** **6**a, Hong et al. examined 14 descriptors of the metal−oxygen bond strength based on 101 intrinsic OER activities of 51 perovskites via factor analysis and linear regression models.^70^ The 14 descriptors included bulk physical properties related to the metal−oxygen bond strength of oxides, such as chemical formalisms (transition‐metal oxidation state), simple models (the Goldschmidt tolerance factor, charge‐transfer energy, or Hubbard U), and experimental structural data (average metal–oxygen bond length). They classified these descriptors into five classes and identified electron occupancy and metal−oxygen covalency to possess the strongest effect on the OER activity. They confirmed that the number of *d* electrons, charge‐transfer energy (covalency), and optimality of *e_g_* occupancy play the important roles, but found that structural factors such as M—O—M bond angle and tolerance factor are relevant as well.

Similarly, as shown in Figure 6b, for graphene‐based single‐atom catalysts, Xu et al. considered elemental electronegativity, the number of nearest‐neighbor N and C atoms coordinated with the metal center, and the correction coefficient in M‐N‐C‐based (M = transition metals) single‐atom catalysts. They statistically defined a more universal descriptor ϕ in regards to Δ*G*_OH*_.^71^

Generally speaking, with the help of machine learning, one enables to correlate descriptors one another and combine them together to develop more powerful predictive descriptors based on current achievements.

## 4 Summary and Outlook

**Figure 7:**
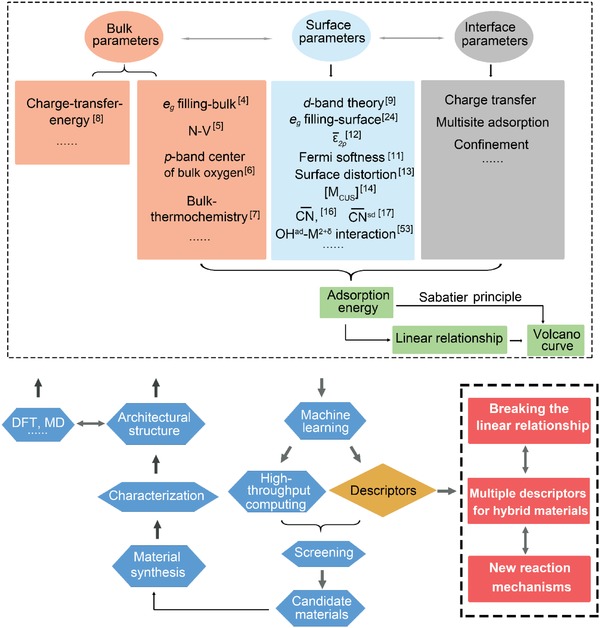
The paradigm to design the next generation descriptors in the application of discovering the catalysts. The current descriptors are summarized, i.e., the quantitative representation of the Sabatier principle except the charge‐transfer energy. Combining experiment and calculation results, the bulk, surface and interface parameters are extracted to rationalize the trends of catalytic performance. Machine‐learning and high‐throughput computing would greatly accelerate the process to identify multiple descriptors and new reaction mechanisms. Discovering the way to break the linear relationship of intermediates would further decrease the overpotential, thus to achieve better activity and selectivity.

In summary, as shown in **Figure** **7**, we have reviewed the achievements for oxygen electrocatalysis descriptors and their applications since the 1960s to date. These descriptors greatly assist in the material screen and optimizations. Nevertheless, the materials in nature including metallic, ionic, covalent, and hydrogen bonds are complicated. It thus remains great challenges to discover more universal, accurate, and measurable descriptors. We conclude by pointing out several important issues and challenges that deserve further investigations:

1) Scaling relationships among the adsorption of oxygen‐containing species greatly facilitate the design of catalysts, but set a theoretical limitation of the overpotential (**≈**0.4 V). Theoretically, Nørskov proposed various schemes to circumvent the scaling relationship for further reduction of overpotentials.^21^ How to experimentally realize them is of great interest.
2) As individual descriptor has the limitation to rationalize the activity trends of all the catalyst materials, development of multiple descriptors might be the key to provide more accurate descriptions of structure‐property features. In addition to that, such multiple descriptors might be extended to the mixed‐phase catalysts. Importantly, machine learning and high‐throughput calculations would play a crucial role to identify such descriptors and new reaction mechanisms.
3) The CHE model acts as one of the pillar stones for the thermodynamic analysis of the ORR/OER process. Nevertheless, the non‐electrochemical process, proton–electron transfer, and recombination of electrons and holes are not included yet in the current CHE model.^72^
4) The microkinetic modeling of surface reactions is a bridge to link quantum‐chemical data with macroscopic behaviors of the systems. Hu et al. proposed the two‐step model which ensures the reaction rates to be determined solely by the chemisorption energies.^73^ Nørskov developed the microkinetic model which provides a better quantitative agreement with the experimental results for ORR than thermodynamic methods.^74^ Although the methods are available, to date, the kinetic process and its application have not drawn enough attention.

## Conflict of Interest

The authors declare no conflict of interest.
